# Online mindfulness-based intervention for women with pregnancy distress: design of a randomized controlled trial

**DOI:** 10.1186/s12884-020-2843-0

**Published:** 2020-03-13

**Authors:** Lianne P. Hulsbosch, Ivan Nyklíček, Eva S. Potharst, Margreet Meems, Myrthe G. B. M. Boekhorst, Victor J. M. Pop

**Affiliations:** 1 Center of Research in Psychological and Somatic disorders (CoRPS), Department of Medical and Clinical Psychology, Tilburg University, P.O. BOX 90153, Warandelaan 2, 5000 LE Tilburg, the Netherlands; 2 UvA minds, academic outpatient (child and adolescent) treatment center of the University of Amsterdam, Amsterdam, the Netherlands; 3 Research Institute of Child Development and Education, University of Amsterdam, Nieuwe Achtergracht 127, Amsterdam, the Netherlands

**Keywords:** Depression, Anxiety, Online intervention, Mindfulness, Pregnancy distress

## Abstract

**Background:**

Psychological distress during pregnancy is common: up to 20% of the childbearing women experience symptoms of depression and anxiety. Apart from the adverse effects on the woman herself, pregnancy distress can negatively affect pregnancy outcomes, infant health, postpartum mother-child interaction and child development. Therefore, the development of interventions that reduce pregnancy distress is very important. Mindfulness-based interventions (MBIs) show promising positive effects on pregnancy distress, but there is a need for randomized controlled trials with sufficient power. Trials on online MBIs, which are readily accessible and not expensive, also show positive effects on stress reduction in non-pregnant populations. Moreover, specific working mechanisms of MBIs remain unclear. The aim of the current study is to test the effectiveness of an online MBI in pregnant women with pregnancy distress, as well as exploring potential working mechanisms.

**Methods:**

The current study is a randomized controlled trial with repeated measures. Consenting women with elevated levels of pregnancy distress will be randomized into an intervention group (MBI) or control group (care as usual) around 12 weeks of pregnancy, with an intended sample size of 103 women in each group. The primary outcome, pregnancy distress, will be assessed via questionnaires at baseline, halfway through the intervention and post intervention in both intervention and control group, and after 8 weeks follow-up in the intervention group. Secondary outcomes are mindfulness skills, rumination and self-compassion, which are also seen as potential working mechanisms, and will be assessed via questionnaires before intervention, halfway through the intervention, post intervention and after 8 weeks follow-up in the intervention group. Tertiary outcome variables are obstetric data and will be collected from the obstetric records for both intervention and control group. Analyses will be based on the intention-to-treat principle. Multilevel regression models for repeated measures (mixed models) will be used to evaluate changes in primary and secondary outcome variables. Tertiary outcomes will be compared between groups using independent t-tests and Chi Square analyses.

**Discussion:**

The trial is expected to increase knowledge about the effectiveness of online MBIs during pregnancy in women with pregnancy distress and to evaluate potential working mechanisms.

**Trial registration:**

ClinicalTrials.gov: NCT03917745, registered on 4 March 2019. Protocol Version 3.0., 20 February 2020.

## Background

Psychological distress during pregnancy is common and affects up to 20% of the childbearing women [1], also in Dutch pregnant populations [2]. Psychological distress is characterized by symptoms of depression and anxiety [3]. During pregnancy, obstetric life events may take place that can negatively affect a woman’s mental health, such as vaginal bleeding, worries about fetal health, abnormal ultrasound outcomes, decreased fetal movements and fear of labor. It is important that pregnant women are capable of coping with these stressful events. Pregnancy distress has been related to poor pregnancy outcomes, including pregnancy induced hypertension, preterm birth and poor fetal health (e.g., low birth weight) [4–8]. Pregnancy distress does not only affect perinatal maternal and infant health, but could also affect the postpartum mother-child interaction [9] and child development [10, 11]. For instance, a review showed that maternal distress during pregnancy is related to mental health problems in the offspring later in life [12]. Most of the research focuses on depression or depressive symptoms during pregnancy, but also anxiety has been related to poor obstetric outcome and developmental and behavioral problems in children [13]. It is clear that it is important to reduce pregnancy distress, for the women’s own mental and physical health, as well as for child development.

Furthermore, recent research by the Central Bureau of Statistics has shown that 15% of the Dutch working population suffers from mental or emotional exhaustion due to work (burnout symptoms) [14]. These symptoms are particularly prevalent among young women between 25 and 35 years old (18%) [14]. Moreover, women between 25 and 45 years are twice as likely to be absent from work compared to men [15]. This difference can partly be explained by physical and psychological problems during pregnancy and postpartum [15]. Nowadays, up to 90% of the pregnant women have a paid job [16]. This means that employee absenteeism is an enormous burden to society, given the fact that stress often results in long-term absence from work. Therefore, strategies and interventions that improve the coping skills of childbearing women regarding perinatal stress may also reduce the burden of absenteeism.

### Mindfulness-based interventions (MBIs)

Mindfulness-based interventions (MBIs) are increasingly being used to decrease symptoms of stress, anxiety and depression [17]. Mindfulness is described as the self-regulation of one’s awareness of experiences in the here-and-now, such as thoughts and feelings, with a curious, open and accepting attitude towards these experiences [18]. This mental state can be trained, leading to positive cognitive and behavioral changes [19, 20]. Research demonstrates positive effects of MBIs, such as reduced levels of stress, anxiety and depression in general population adults, and in those with specific somatic conditions such as diabetes [20–22]. The specific working mechanisms in MBIs remain to be elucidated. Possibly, alterations in mindfulness skills, rumination and self-compassion are associated with or mediate the effect of MBIs on stress, anxiety and depression [23–25]. Rumination is characterized by repetitive thoughts about causes, situational factors and consequences of one’s negative emotional experience [26]. Self-compassion is described as being warm, open and understanding toward one’s own suffering or feelings of inadequacy, instead of being judgmental or avoidant towards these feelings [27].

Through mindfulness training, people learn to pay attention to their thoughts, sensations, and emotions that arise, and accept them for what they are, without ‘losing themselves’ in these thoughts and feelings [28]. These skills are helpful to pregnant women who experience anxiety about pregnancy and childbirth. Furthermore, a recent study showed that a low score on maternal mindfulness skills was related to a higher risk of her neonate’s low birth weight, controlled for the effect of depression [29].

Several recent reviews have been published evaluating the effect of MBIs during pregnancy on maternal mental health [30–33]. The reviews all concluded that MBIs during pregnancy show promising positive effects on maternal distress, including depression and anxiety. However, the overall level of evidence is weak, largely due to small sample sizes. Therefore, there is a clear need for high-quality research on this subject to examine the effectiveness of MBIs during pregnancy. Specifically, there is a need for randomized controlled trials with sufficient power.

### MBIs delivered via internet

Because of the large number of pregnant women confronted with mental health problems annually (20% [1] of 170.000 [34] pregnancies per year in the Netherlands) it is important to develop interventions that are effective, readily accessible and inexpensive. E-health interventions for mental health problems in general have become increasingly popular during the last decades. These often web-based interventions are inexpensive and accessible to most of the population. Almost all women of the childbearing age are familiar with internet [35].

First trials on MBIs delivered via internet for non-pregnant women show positive effects on stress reduction. A meta-analysis of 15 RCTs in non-pregnant women showed that online MBIs had a small significant positive effect on depression, anxiety, well-being and mindfulness, and a moderate significant positive effect on stress, with effect sizes being 0.29, 0.22, 0.23, 0.32 and 0.51 respectively, calculated with Hedge’s g [36].

### Objectives and hypotheses

The aim of the current study is to test the effectiveness of an online MBI in pregnant women with pregnancy distress. The primary outcome is pregnancy distress, i.e. symptoms of depression and anxiety. Secondary outcomes are mindfulness skills, rumination and self-compassion, which are also seen as potential working mechanisms. Tertiary outcomes are obstetric data such as gestational age at birth, use of anesthesia, mode of delivery and birth weight. It is hypothesized that pregnancy distress in women who participate in the 8-week online MBI course (intervention group) will be significantly reduced compared to a control group, who does not complete this course and receives care as usual. With regard to the secondary outcomes, it is hypothesized that the intervention will lead to improvements in mindfulness skills and self-compassion, and a decrease in rumination for the intervention group. We will investigate whether the potential working mechanisms, i.e. mindfulness skills, rumination and self-compassion, are associated with a reduction in pregnancy distress in the intervention group.

## Method/design

### Design and setting

The current study is a parallel group, two-arm, superiority randomized controlled trial (RCT) with equal allocation of participants and a repeated measures design. Table 1 shows the overall study design. The trial is part of a large longitudinal cohort study among pregnant women (The Brabant Study). Within the Brabant Study up to 4000 pregnant women are followed from 12 weeks of gestation until 8 to 10 weeks postpartum. The current trial targets a specific sub-group of pregnant women with symptoms of psychological distress, specifically depression and anxiety and is conducted in the South-East part of North-Brabant in the Netherlands. The trial is registered at clinicaltrials.gov (2018-CDE-9318) and was approved of by the Ethics Committee of the University of Amsterdam. Any trial amendments will be approved of by the Ethics Committee of the University of Amsterdam before implementation and will be reported to the trial registry.

**Table 1:**
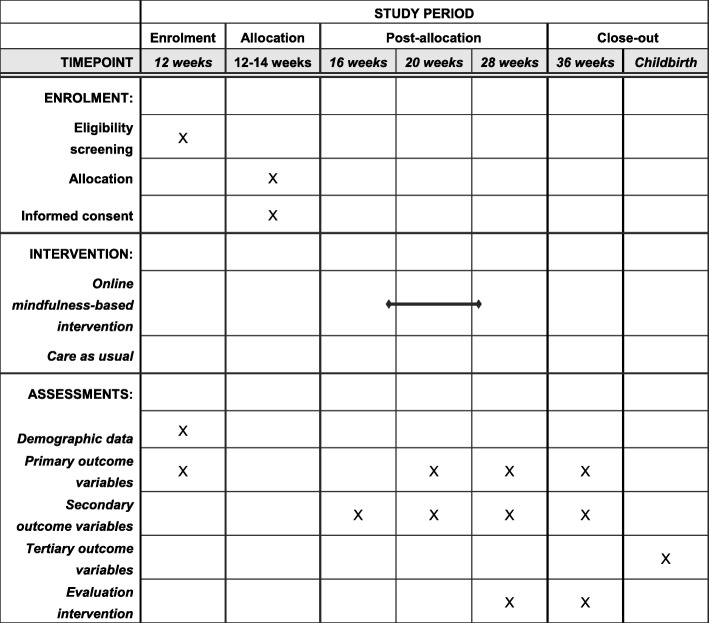
SPIRIT Schedule of enrolment, intervention and assessments during pregnancy

*Note*: 12 weeks, baseline; 16 weeks, before intervention; 20 weeks, halfway through the intervention; 28 weeks, post intervention; 36 weeks, after 8 weeks follow-up

### Participants

For the Brabant Study, women will be recruited by community midwife practices during their first antenatal visit. Inclusion criteria for participation in the cohort study are: pregnant women (18+ y) with the first antenatal visit before 14 weeks gestation and an understanding of the Dutch language. Exclusion criteria are: multiple pregnancy (or higher order pregnancy), known endocrine disorder before pregnancy (other than thyroid function problems), diabetes type I, rheumatoid arthritis, severe psychiatric disease (schizophrenia, borderline personality disorder or bipolar disorder), HIV, drug or alcohol addiction problems, or any other disease resulting in treatment with drugs that are potentially adverse for the fetus and need careful follow-up during pregnancy, and no access to the internet.

### Measures

#### Primary outcome measure

##### Pregnancy distress

Symptoms of depression during pregnancy are measured with the 10-item Edinburgh Depression Scale (EDS), which is validated among both Dutch postpartum women [37, 38], and pregnant women [39]. The EDS consists of three facets: *anhedonia*, *anxiety* and *depression*, of which *anhedonia* and *depression* assess different concepts of depression [40]. The EDS is a reliable instrument to screen for depression in each trimester of pregnancy. The Cronbach’s alpha’s are 0.82, 0.83 and 0.84 per trimester respectively [39]. The items are scored on a four-point Likert scale ranging from 0 to 3. The total score of the EDS ranges from 0 to 30, with higher scores reflecting more depressive symptoms. Trimester-specific cut off points were determined, lower than the commonly applied cut off in the postpartum period. During gestation a cut off of 11 for depression at the first trimester has been described, and a cut off of 10 in the second and third trimester [39]. In the current study, not only women with depression but also women with depressive symptoms will be included. For the cut off score in the current study, we used data of a similar large cohort study in the South-East part of North-Brabant in the Netherlands, the Holistic Approach to Pregnancy and the first Postpartum Year (HAPPY) study [41]. The cut off score is defined as the upper quartile of a sample of approximately 2000 women at 12 weeks of pregnancy of the HAPPY study. This results in a cut off score of > 7.

Furthermore the negative affect (NA) subscale of the Tilburg Pregnancy Distress Scale (TPDS) will be used [42]. This 11-item subscale of the TPDS measures worry symptoms about pregnancy and labor, using a four-point Likert scale ranging from 0 (rarely/never) to 3 (very often). The total score of the NA subscale of the TPDS ranges from 0 to 33, with higher scores indicating higher levels of pregnancy-related distress. The TPDS has been validated in Dutch pregnant women with an overall Cronbach’s alpha of 0.78 and 0.81 for the NA subscale at different trimesters [42]. The TPDS has been shown to be a valid and reliable instrument, with adequate psychometric properties [43]. The cut off score for the current study is defined as the upper quartile of the NA subscale scores of women participating in the above described sample of the HAPPY study [41], which results in a cut off score of > 9.

#### Secondary outcome measures

##### Mindfulness skills

Mindfulness skills will be assessed using the Three Facet Mindfulness Questionnaire-Short Form (TFMQ-SF) [44]. Only three subscales (a*cting with awareness*, *non-judging* and *non-reacting*) of the original Five Facet Mindfulness Questionnaire (FFMQ) [45, 46] were found to significantly predict psychological symptoms [47]. The three subscales each contain five items and are answered on a five-point Likert scale ranging from 1 (never or very rarely true) to 5 (very often or always true), with a total score ranging from 15 to 75. Higher total scores indicate better mindfulness skills. The TFMQ-SF has been shown to be a valid instrument in Dutch pregnant women with adequate psychometric properties and Cronbach’s alphas of 0.87 (*acting with awareness*), 0.84 (*non-judging*) and 0.81 (*non-reacting*) [44].

##### Rumination

The rumination subscale of the Rumination-Reflection Questionnaire (RRQ) will be administered to assess rumination [48]. This 12-item subscale of the RRQ uses a five-point Likert scale ranging from 1 (strongly disagree) to 5 (strongly agree). The total score of the rumination subscale ranges from 12 to 60, with higher scores indicating more rumination. The RRQ has good convergent and discriminant validity, and a Cronbach’s alpha of 0.90 for the rumination subscale in Dutch samples [49].

##### Self-compassion

Self-compassion will be measured with the 12-item Self-Compassion Scale-Short Form (SCS-SF) [50], which is derived from the Self-Compassion Scale (SCS) [27]. The six facets of the SCS-SF are: *self-kindness*, *self-judgement*, *common humanity*, *isolation*, *mindfulness* and *over-identification*. All items are measured on a seven-point Likert scale ranging from 1 (almost never) to 7 (almost always), with a total score ranging from 12 to 84. Higher scores on the SCS-SF reflect greater levels of self-compassion. The validated SCS-SF has adequate internal consistency with a Cronbach’s alpha of 0.87 in Dutch samples [50].

#### Tertiary outcome measures

##### Obstetric data

As part of the Brabant Study, data on pregnancy and delivery will be collected from the community midwife and/or obstetrician, by extracting these data from the obstetric form. This concerns obstetric data such as gestational age at birth, use of anesthesia, mode of delivery and birth weight.

#### Additional outcome measures

##### Demographic data

As part of the Brabant Study, demographic data such as age, marital status, ethnicity, education, working status and number of the children in the home will be collected via online questionnaires.

##### Evaluation of the intervention

Questions for evaluation of the online MBI course are based on the stress reduction program evaluation, developed at the Center for Mindfulness of the University of Massachusetts medical school. The questions include the number of sessions the women have completed and if applicable, the reasons why they did not finish the course; how much time they spent on practicing mindfulness, during the course and at the present moment; experiences of each session and the course as a whole; intention to continue practicing mindfulness after the course; possible changes in taking care of themselves, recognizing and dealing with stress, dealing with emotions and contact with others; possible negative effects of the course (which are not anticipated).

### Procedure

Within the Brabant Study, women will receive their first questionnaire (related to obstetrics and mental health) at 12 weeks of pregnancy via internet. Women with a score above the cut off on the EDS (> 7) and/or the NA scale of the TPDS (> 9) will be randomly allocated to the intervention or control group of the current study. Moreover, women with depressive symptoms that are too severe will not be included in the trial. These women are defined by an EDS score > 18. Also, women who did not answer the last question of the EDS (“The thought of harming myself has occurred to me”) with “never” will not be included in the trial. Instead, author LH will send an e-mail to encourage these women to seek help with their general practitioner (GP).

LH monitors the scores on the EDS and TPDS and, when a woman scores above cut off, passes on the corresponding participant number to author MM who has no further involvement in the practical recruitment, enrolment and assessment of patients in the trial. MM will refer to a list of random numbers of 1 (intervention) and 2 (care as usual), which will be created by MM using the Statistical Package of Social Science (SPSS), version 24. The random numbers will be sequentially assigned to subsequent participants. MM will inform LH about the allocation by e-mail and will archive the allocations in a secured document on her computer. LH will document the allocation in the general inclusion database, which will be checked by MM. LH will contact and invite the women in the intervention group by e-mail to participate in the online MBI course. The control group will receive care as usual and will fill out the questionnaires according to the Brabant Study. Women in both the intervention and control group have already provided written informed consent for participation in the Brabant Study. The above described procedure of randomization will ensure objectivity of the researcher and will eliminate bias in the participants’ group allocation. Blinding of participants or researchers is not possible due to the study design.

The women allocated to the intervention group will receive an information letter about the trial and the online MBI course by e-mail. Women will be asked to respond by e-mail whether they want to participate. When women agree to participate, they will sign a full written informed consent, which will be sent by post. After signing the informed consent, women will fill out questionnaires via internet, which will be around 16 weeks of pregnancy. Besides the standardized questionnaires, women will be asked whether they have had any previous experiences with mindfulness. Subsequently, women will start the 8-week online MBI course, followed between 16 and 28 weeks of pregnancy. Women in both the intervention and control group are not prohibited to partake in any other pregnancy course. At 28 weeks of pregnancy (post intervention) women in both groups will be asked whether they took part, or are still taking part, in adjunctive pregnancy courses, to be able to control for this variable in statistical analyses.

Participating women fill out online questionnaires at different time points during pregnancy as shown in Table 2. Women in the intervention and control group fill out the questionnaires of the Brabant Study at baseline (12 weeks of pregnancy), halfway through the intervention (20 weeks of pregnancy) and post intervention (28 weeks of pregnancy). Moreover, women in the intervention group fill out extra questionnaires before intervention (16 weeks of pregnancy), halfway through the intervention, post intervention and after a follow-up period of 8 weeks (36 weeks of pregnancy).

**Table 2:** Measurements and time points

	Concept	Questionnaire	Measurement time points											
			12 weeks		16 weeks		20 weeks		28 weeks		36 weeks		Childbirth	
			I	C	I	C	I	C	I	C	I	C	I	C
Primary	Pregnancy distress	EDS	X	X			X	X	X	X	X			
	Pregnancy distress	TPDS (NA)	X	X			X	X	X	X	X			
Secondary	Mindfulness skills	TFMQ-SF			X				X		X			
	Rumination	RRQ			X				X		X			
	Self-compassion	SCS-SF			X				X		X			
Tertiary	Obstetric data	Non-standardized											X	X
	Demographic data	Non-standardized	X	X										
	Evaluation intervention	Non-standardized							X		X			

*Note*: 12 weeks, baseline; 16 weeks, before intervention; 20 weeks, halfway through the intervention; 28 weeks, post intervention; 36 weeks, after 8 weeks follow-up; I, intervention group; C, control group; EDS, Edinburgh Depression Scale; TPDS, Tilburg Pregnancy Distress Scale; NA, negative affect; TFMQ-SF, Three Facet Mindfulness Questionnaire Short Form; RRQ, Rumination Reflection Questionnaire; SCS-SF, Self-Compassion Scale Short Form; MBI, mindfulness-based intervention

### Intervention

The online MBI course is based on existing protocols of the Mindfulness-Based Stress Reduction (MBSR) as described by Kabat-Zinn [51] and Mindfulness-Based Cognitive Therapy (MBCT) as described by Segal et al. [52]. It consists of eight one-hour sessions including psycho-education about the mechanisms of stress, coping, and relaxation, especially related to pregnancy, practicing mindfulness skills (i.e. mindful breathing, mindful moving, observing and letting go of thoughts and emotions with a non-judgmental attitude), sharing experiences, and completing home assignments. The sessions have been developed specifically for pregnant women and acknowledge pregnancy-related distress. Table 3 shows a short overview of the online MBI course. The website of the course can be viewed at https://www.ontspannenzwanger.nl/. Within the current study, the course will be offered free of charge to participating women. As far as we know, there are no known risks of the intervention to pregnant women.

**Table 3:** Content of online MBI course ‘Ontspannen zwanger’ (In English: ‘Relaxation in pregnancy’)

Week	Session
1	**Stress and Mindfulness** Exercises in online session: bodyscan Home assignments (daily): bodyscan, routine activity, activity awareness
2	**Dealing with obstacles, a different view** Exercises in online session: mindful breathing Home assignments (daily): bodyscan, mindful breathing, logbook pleasant events
3	**The body and the senses** Exercises in online session: mindful yoga, 3 min breathing space Home assignments (daily): bodyscan/yoga, 3 min breathing space, routine activity, logbook unpleasant events
4	**Thoughts** Exercises in online session: observing thoughts, mindful walking Home assignments (daily): bodyscan/yoga/sitting meditation, 3 min breathing space, routine activity
5	**Emotions** Exercises in online session: working with emotions Home assignments (daily): sitting meditation/bodyscan/yoga, 3 min breathing space, routine activity, logbook stressful communication
6	**Communication and awareness** Exercises in online session: conscious communication, mindful breathing and open awareness, sounds and open awareness Home assignments (daily): sitting meditation/bodyscan/yoga, 3 min breathing space, routine activity, conscious communication
7	**Take good care of yourself and your baby** Exercises in online session: free moving meditation and breathing towards belly and pelvis Home assignments (daily): sitting meditation/bodyscan/yoga, 3 min breathing space, routine activity, energy balance (once a week), planning of pleasant activities
8	**The beginning of a new way of life** Online session: stress signals and strategies, tips for mindfulness practice in daily life

*Note:* MBI, mindfulness-based intervention

Personal feedback of the participants in a recent related study [53] showed that women with toddlers, during the course, preferred weekly contact (via internet) with a trainer to encourage following the weekly sessions and to ask for possible problems that participants might encounter. In the current study LH, a certified mindfulness trainer, will review questions of participants and will send weekly reminders to positively encourage the participating women to follow the course and to ask for help when they need it. This online mindfulness trainer is important to facilitate feedback and improve the process of successfully completing the course. Furthermore, a meta-analysis of web-based interventions showed that the effectiveness of an intervention is higher when the participants have the possibilities to communicate with a trainer [36].

### Sample size calculation

Within the Brabant Study, 4000 women will be included during a period of 2 years [53]. In a recent related study, an online mindful parenting training for parental stress was examined for feasibility. During this study, it has become clear that of all eligible women (20%), 35% consented to participate with a drop-out rate of 10–15% (Meems M, Hulsbosch LP, Hendricx Riem MME, Meyers C, Pronk T, Broeren MAC, Nabbe KCAM, Oei G, Pop VJM: The Brabant Study: design of a large prospective perinatal cohort study among pregnant women investigating obstetric outcome from a biopsychosocial perspective, submitted). In the Brabant Study, 20% of 4000 will be eligible for intervention, meaning 800 women, with 400 women in each group. Of these 400 women in the intervention group, it is reasonable to expect that 35% of 400 = 140 women will consent to participate in the intervention. Considering a drop-out rate of 15%, 119 women in the intervention group will complete all questionnaires.

The total number of women that have to be included in current study has been calculated using G-power. Based on a medium effect size (Cohen’s d = 0.5) of a time by group interaction in a MANOVA with three time points and power = 0.90, the calculation results in a total sample size of 206 women, with 103 women in each group. Therefore, the number needed for the trial can be met in 2 years of inclusion, also including an expected attrition of about 10–15%.

### Statistical analyses

The baseline characteristics of completers and drop outs during follow-up will be compared by means of an independent t-test for continuous data and by Chi Square analysis for categorical data. Any significant differences (*p* < 0.05) in baseline characteristics between intervention and control group will be controlled for in the subsequent analyses. All analyses will be based on the intention-to-treat principle.

For the main analyses, multilevel regression models for repeated measures (mixed models) will be used to evaluate changes in primary outcome variables across time between intervention and control group and to evaluate changes in secondary outcomes across time in the intervention group. Temporal associations between change in secondary outcomes that may be potential mechanisms and change in primary outcomes will be examined by linear regression analyses, in which decrease in primary outcome scores are predicted by earlier changes in secondary outcome scores (and vice versa to control for potential reversed effects). Tertiary outcomes will be compared between intervention and control group using independent t-tests for continuous data and Chi Square analyses for categorical data.

### Data management

LH will be responsible for data collection under supervision of the corresponding author VP, who is the principal investigator and responsible for the project management in general. All data on paper will be stored in a locked, secure area at Tilburg University. Digital data will be stored in a project data package, according to the data management guidelines of Tilburg University. Patient identifiable data (e.g., contact information and informed consent forms) will be stored separately from anonymized data. Patient confidentiality will be protected in line with the ethics guidelines of Tilburg University and the University of Amsterdam.

Data will be analyzed by LH after trial completion and no interim analyses will be done. Data will not be released before completion of the trial. Data (anonymized) will be shared directly after publication with restricted access, which means that data may be made available on reasonable request from the corresponding author VP.

### Trial status

As of 20 February 2020, the study is recruiting participants, with 28 enrolled so far.

## Discussion

Current study’s objective is to test the effectiveness of an online MBI in pregnant women with pregnancy distress. For this purpose, we will compare an intervention group with a control group who receives care as usual. The primary outcome is a decrease of pregnancy distress, i.e. symptoms of depression and anxiety. Secondary outcomes are potential working mechanisms, such as mindfulness skills, rumination and self-compassion. Tertiary outcomes are obstetric data like gestational age at birth, use of anesthesia, mode of delivery and birth weight in relation to changes of distress symptoms.

The current study has both strengths and limitations. Strengths of the study include the randomized controlled design and the expected sample size of 119 women in the intervention group. The current study is part of the Brabant Study, a large longitudinal birth cohort study among 4000 pregnant women, which makes inclusion of eligible women easily feasible. A limitation of the study is that the secondary outcome variables, the potential working mechanisms, can only be explored in the intervention group. The control group will fill out the questionnaires of the Brabant Study only, in which mindfulness skills, rumination and self-compassion are not measured.

Because there is a need for randomized controlled trials with sufficient power evaluating the effect of MBIs during pregnancy on maternal mental health [30–33], the current study is clearly of added value, and is expected to increase knowledge about the effectiveness of online MBIs during pregnancy in women with pregnancy distress. Furthermore, the current study will contribute to the knowledge of potential working mechanisms in MBIs, since reviews suggest changes in mindfulness skills, rumination and self-compassion to be associated with or to mediate the effect of MBIs on stress, anxiety and depression [23–25], but no definite conclusions can be drawn yet [23].

An essential part of the study is that the outcome of the current study (e.g., an online MBI with proven effectiveness to reduce distress in pregnant women) should be disseminated over the whole country. As a consequence, we will focus on the distribution of study results and derived knowledge through the following five different approaches. First, all steps that are necessary to implement this program within perinatal care will be carefully registered during the study, taking into account problems encountered during the set-up of the program. This will result in a general guidelines book at the end of the study. Special attention will be given to obstacles in the procedure and obstructive and promoting factors. Second, in the local area, the consortium structure and its collaborators (including midwifes, obstetricians and maternity care-workers) will be used to inform all healthcare workers about the online MBI course. For this purpose, we have a budget to further reinforce the set-up of a center of knowledge and excellence within the local consortium. This will contribute to setting up an infrastructure that will persist in the future to improve perinatal care. The members of the local consortium will be visited and instructed how to implement the course in daily practice. Third, other consortia will be invited to implement the program into regular care. Fourth, in the Netherlands, there are up to 100 GP caregiver groups who coordinate health care provided by the GP nurse (in Dutch: praktijkondersteuner huisartsen (POH)). One of these POHs is the mental health POH. The mental health POH is currently involved in diagnostic and (simple) therapeutic trajectories for patients with mental health problems. The mental health POH is also the designated person within primary care to inform patients about web-based interventions. Because midwives still have no access to these POHs, except after referring to the GP, we will invest in setting up programs to incorporate mental health care to pregnant women within the POH mental health care system. During the period of implementations, collaborative steps will be undertaken with the organization for POHs (in Dutch: Praktijkondersteuning Zuid-Oost-Brabant (PoZoB)) to implement this POH in perinatal mental healthcare. Principal investigator VP was one of the co-founders of PoZoB, and as the scientific adviser of PoZoB, he implemented the general mental health care program for the POH within PoZoB. Finally, it should be noticed that our society is increasingly familiar with the internet as a means to get access to all kinds of facilities. This means that every pregnant woman can get access to this online MBI by logging in into the website https://www.ontspannenzwanger.nl/. Once the effectiveness has been proven, the website will be more intensively promoted on the internet. Appropriate steps will be undertaken to make sure the website is easily accessible through the most common search engines.

Data output of current study will be published in international peer reviewed scientific journals to distribute the findings internationally. The first results of current study will become available in 2022.

## Data Availability

Datasets generated during the current study may be made available on reasonable request from the corresponding author.

## Abbreviations

EDS: Edinburgh Depression Scale
FFMQ: Five Facet Mindfulness Questionnaire
GP: General practitioner
HAPPY: Holistic Approach to Pregnancy and the first Postpartum Year
MBCT: Mindfulness-Based Cognitive Therapy
MBI: Mindfulness-based intervention
MBSR: Mindfulness-Based Stress Reduction
NA: Negative Affect
POH: Praktijkondersteuner huisartsen (general practitioner health care nurse)
PoZoB: Praktijkondersteuning Zuid-Oost-Brabant (organization for general practitioner health care nurses)
RCT: Randomized controlled trial
RRQ: Rumination-Reflection Questionnaire
SCS-SF: Self-Compassion Scale-Short Form
SPIRIT: Standard Protocol Items Recommendations for Interventional Trials
SPSS: Statistical Package of Social Science
TFMQ-SF: Three Facet Mindfulness Questionnaire-Short Form
TPDS: Tilburg Pregnancy Distress Scale
